# Investigating the component structure of the Health of the Nation Outcomes Scales for people with Learning Disabilities (HoNOS-LD)

**DOI:** 10.1177/00207640251323819

**Published:** 2025-03-12

**Authors:** Jon Painter, Kiran Purandare, Joanne McCabe, Ashok Roy, Rohit Shankar

**Affiliations:** 1 Sheffield Hallam University, UK; 2 Central and Northwest London NHS Foundation Trust, UK; 3 University Hospital Coventry and Warwickshire, UK; 4 Coventry and Warwickshire Partnership Trust, Birmingham, UK; 5 Cornwall Intellectual Disability Equitable Research (CIDER), Cornwall Partnership NHS Foundation Trust, Truro, UK; 6 Cornwall Intellectual Disability Equitable Research (CIDER), University of Plymouth Peninsula School of Medicine, Truro, UK

**Keywords:** HoNOS-LD, Health of the Nation Outcome Scales, Principal Component Analysis

## Abstract

**Background:**

Outcome measurement is increasingly recognised as a vital element of high-quality service provision, but practice remains variable in the field of intellectual disabilities. The Health of the National Outcome Scales for people with Learning Disabilities (HoNOS-LD) is a widely used Clinician Reported Outcome Measure in the UK and beyond. Over its 20-year lifespan, its psychometric properties have been frequently investigated. Multiple dimensionality reduction analyses have been published, each proposing a different latent structure.

**Aim:**

To analyse a set of HoNOS-LD ratings to test its internal consistency, to identify the optimal number of latent variables, and to propose the items that group together in each domain.

**Methods:**

A Principal Component Analysis of 169 HoNOS-LD ratings was performed to produce an initial model. The component loadings for each HoNOS-LD item were then examined, allowing the model to be adjusted to ensure the optimal balance of statistical robustness and clinical face-validity.

**Results:**

HoNOS-LD’s internal consistency (18 items) was ‘acceptable’ (Cronbach’s alpha = 0.797). On excluding three items that had no bivariate correlations with the other 15 items internal consistency rose to ‘good’ (Cronbach’s alpha = 0.828). The final, four-component solution, using the 15 items possessed good internal reliability.

**Conclusion:**

HONOS-LD statistical properties compared favourably to the other published latent structures and adheres to the tool’s rating guidance. The four-component solution offers an acceptable balance of statistical robustness and clinical face validity. It provides advantages over other models in terms of internal consistency and/or viability for use at a national level in the UK.

## Introduction

For many years, measuring the impact of mental healthcare has frequently been limited to the use of Clinician Reported Outcome Measures (CROMS) but their adoption into routine clinical practice has been variable, with many psychiatrists expressing concerns and a reluctance to engage with this agenda (Gilbody et al., 2002). More latterly, interest has grown in the use of Patient Reported Outcome Measures (PROMs) however, even their use has, according to Wolpert (2014) created concern for some clinicians who feel their introduction has added to bureaucracy without offering any benefits to patients or staff. In addition to the limitations of each individual approach, measuring health and social care outcomes for people with intellectual disabilities and/or autism is more challenging than many other areas of healthcare (Bertelli et al., 2022), necessitating a blended approach that incorporates both CROMS, PROMS and also Patient Reported Experience Measures (PREMS).

The Health of the Nation Outcome Scales for people with Learning Disabilities (HoNOS-LD) (Roy et al., 2002) is one of a relatively small number of CROMs that is widely used in services for people with intellectual disabilities in the UK (Hunt et al., 2023). The HoNOS-LD (Roy et al., 2002) is one of a ‘family’ of similar measures, each intended to capture health and social care outcomes of a different patient group. It was developed after the original (working aged adult, mental health) version – HoNOS (Wing et al., 1998) was found to have limitations when used in specialist intellectual disability services (Ashaye et al., 1997). The HoNOS-LD is intended to be a brief, holistic measure that is acceptable to a range of professionals working with people with intellectual disability in routine clinical practice. Following development and original testing, its psychometric properties have been confirmed in numerous studies (Kumar et al., 2024; Tenneij et al., 2009).

A key aspect of any scale’s validity is the extent to which its items are internally consistent, as well as how they can be grouped/reduced (Boateng et al., 2018). From a review of the literature, we identified four community-based studies that have statistically derived sub-scales for the HoNOS-LD and one inpatient study that used clinical opinion alone to propose domains. Across these studies there is variation in both the number of domains deemed optimal, as well as which items group together in the domains. This variation limits the benefits of a nationally mandated tool as different organisations will be using different domains (sub-scales) to report outcomes, making direct comparisons problematic.

The aim of this study was to therefore to analyse a set of HoNOS-LD ratings to test its internal consistency, to identify the optimal number of latent variables, and to propose the items that group together in each domain.

## Method

### Measure

HONOS-LD has 18 items, each one rating the most severe occurrence of a particular phenomenon in the previous 4 weeks, on a 0 to 4 scale (no problems – very severe problems). The original validation study (Roy et al., 2002) was based on the ratings of 372 individuals with intellectual disabilities by 364 raters from six different healthcare professions who work with people with intellectual disabilities. In this, the tool was reported to have good inter-rater reliability, sensitivity to change and to correlate well with other validated measures. The HoNOS-LD can be accessed here: HoNOS-LD glossary.pdf

### Data Analysis

**Figure 1.**
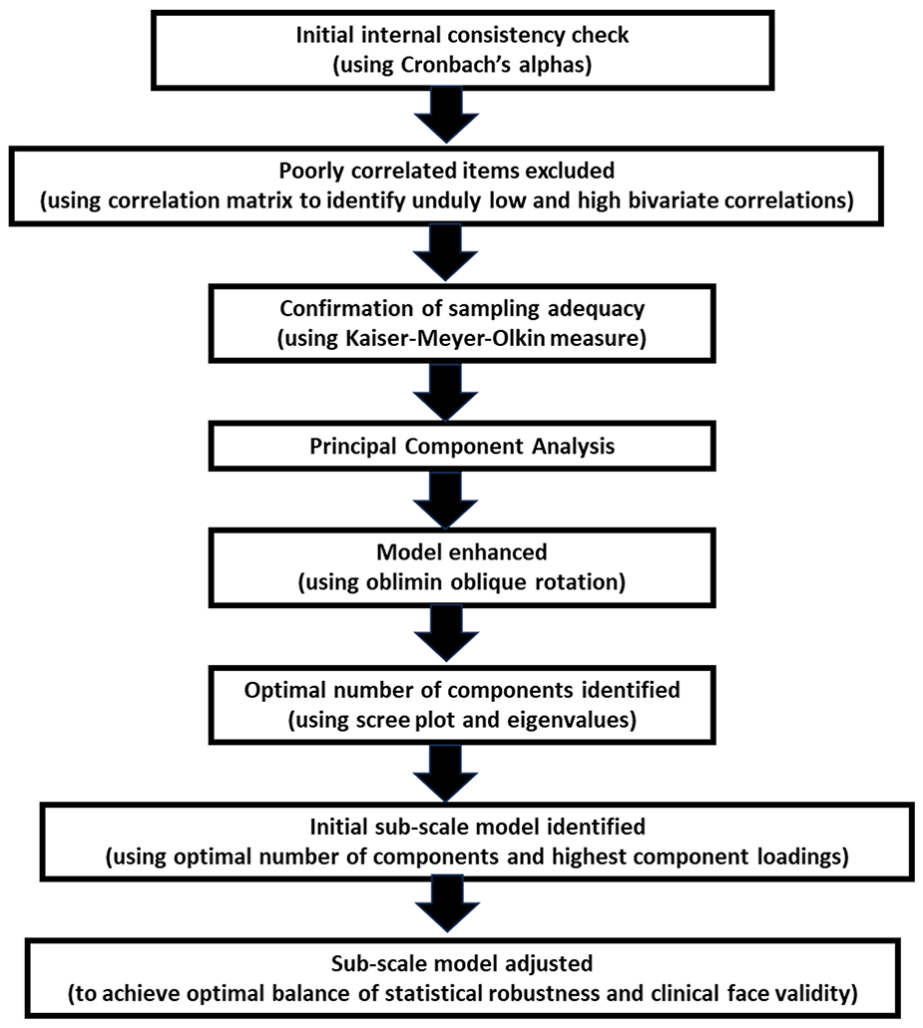
Data analysis flow chart.

The complete sets of HoNOS-LD ratings were exported into SPSS (Version 26) for analysis purposes. Internal consistency was confirmed using Cronbach’s alpha before a correlation matrix was produced to identify any items with correlations lower than 0.3 or above 0.9. Having excluded these outlying items (Field, 2009), sampling adequacy for the remaining individual items and the overall data set were confirmed using the Kaiser–Meyer–Olkin (KMO) measure. A Principal Component Analysis (PCA) was then performed with direct oblimin oblique rotation as there was a theoretical case to be made that scales which capture aspects of the human nature will inevitably be related to some degree. The optimal number of components was determined with reference to all eigenvalues over Kaiser’s threshold of 1.0 and to a scree plot of eigenvalues against components. The PCA was then re-run with that number of components specified. An initial component solution was created based solely on the highest component loading for each item. Cronbach’s alpha values were calculated for each of the four potential HoNOS-LD subscale scores identified through the PCA. Where internal consistency of components was low, and/or where items loaded onto multiple components, this information was borne in mind when item groupings were adjusted to ensure the final component solution had the optimal balance of statistical robustness and clinical face validity. Figure 1 shows the stages of the data analysis we undertook.

### Ethics

Ethical approval for the project was obtained from Sheffield Hallam university (ID: ER59802899). This study was a secondary analysis of data that had been routinely gathered as part of a previously published study (Abraham et al., 2022). In brief, that study retrospectively extracted a set of routinely recorded pseudonymised clinical data (including HoNOS-LD ratings) from inpatient records. Individual patient consent was, therefore, not required for the original project, nor this secondary analysis.

## Results

### Sample

The HoNOS-LD ratings used in this secondary data analysis study were from the records of 169 patients admitted to one specialist intellectual disability unit in London over an 8-year period (Abraham et al., 2022). The study reported 49 (29%) individuals to be female and 120 (71%) as male, with an overall mean age of 30.9 years. In the study cohort 98 (58%) had a diagnosis of mild intellectual disability whilst the remaining 71 (42%) had moderate – severe intellectual disability. Of the total sample, 105 (62%) had comorbid mental health diagnoses, 85 (50%) had autism spectrum disorder, and 127 (75%) exhibited behaviours of concern. In total there were three raters. All raters had received the same training package on use of the HoNOS-LD.

### Analysis

Internal consistency of the HoNOS-LD, when all 18 items were included, was found to be ‘acceptable’ (Cronbach’s alpha = 0.797). Three items had no bivariate correlations with any of the other items that fell within the 0.3 to 0.9 range recommended by Field [11]. These were item eight problems with hallucinations and delusions; item 12 Physical problems; item 13 Seizures. Whilst these have clinical utility in capturing the needs of some individuals, by excluding these outliers, the internal consistency of the remaining 15 items rose to ‘good’ (Cronbach’s alpha = 0.828).

Having identified (and excluded) the items which were unlikely to be measuring the same underlying construct as the majority of the HoNOS-LD items, the Kaiser–Meyer–Olkin (KMO) measure verified the overall sampling adequacy for a Principal Component Analysis, KMO = 0.795, as well as for the individual items (all KMO values above 0.572).

An initial analysis (PCA with direct oblimin oblique rotation) was performed to obtain eigenvalues for each component in the data. Four components had eigenvalues over Kaiser’s criterion of 1 and together explained 62.25% of the total variance. The scree plot showed inflexions that could justify the retention of two, three, or four components. However, the two and three component solutions yielded too many multiple loadings to allow the HoNOS-LD items to be grouped with any confidence therefore, given Kaiser’s eigenvalue criterion, all four components were ultimately retained. Table 1 shows the component loadings above 0.384, after rotation, which is the level for this sample size recommended (Stevens, 2002). The shading illustrates the solution based solely on the highest component loadings for each item.

**Table 1.** Summary of PCA direct oblimin oblique rotated components based solely on each item’s highest component loading.

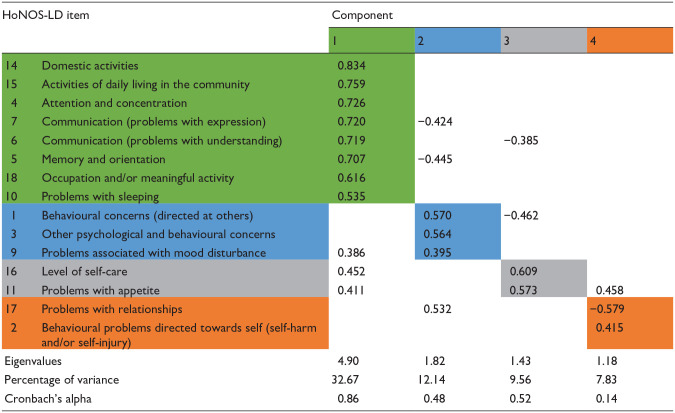					

Table 1 also shows the Cronbach’s alpha values for each of the four potential HoNOS-LD subscale scores identified through the PCA from which it is apparent that the internal consistency of components two and four were low. Following adjustment of items with multiple component loadings to enhance clinical face validity (Table 2), internal consistency of components also improved which confirmed the optimal balance between clinical and statistical validity had been reached. After this, each component was given a title that represented the nature of the items it included. These titles were: Cognition and functioning (items 5, 4, 10, 7, 6, 15, 14, 18); Interpersonal behaviours (items; 1, 3, 9, 17) Self-caring (items 16, 11); and Self-injurious behaviours (item 2).

**Table 2.** Summary of PCA direct oblimin oblique rotated components after refinement to balance clinical and statistical validity.

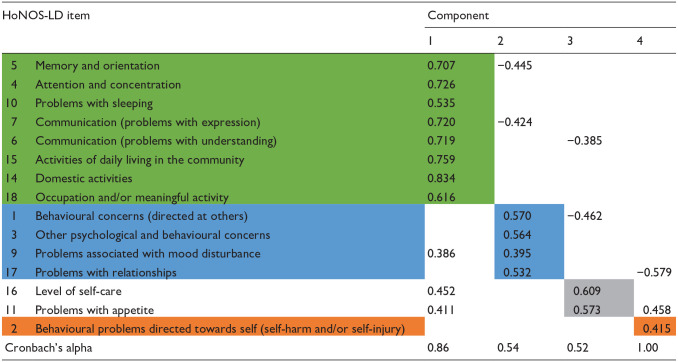					

## Discussion

The results of our principal component analysis, using a set of inpatient HoNOS-LD ratings, yielded a four-component solution which had acceptable statistical properties as well as clinical face validity (see Table 2). To our knowledge, the only other published study undertaken using inpatient HoNOS-LD data (Hillier et al., 2010), used clinical opinion alone, meaning the statistical robustness and coverage of their solution was not stated.

Four other studies have used a range of statistical methods to identify the sub-scale structure of HoNOS-LD however, these were all generated from ratings undertaken in community settings. In addition, only one of these models (Hunt et al., 2023) adhered to the tool’s rating guidance for item 3 (whereby only the highest of its five picklist options is recorded), meaning the other solutions would be problematic to apply at a national level as the mandated dataset only permits the most severe rating to be submitted. In this regard, our results make a helpful contribution to the existing knowledge base.

Table 3 summarises the solutions proposed by each published study we identified. Shading has been added to highlight consistencies in the way the HoNOS-LD items have been grouped across studies. From this it is clear that there is no universally accepted solution however, items 4 (attention and concentration), 5 (memory and orientation), 6 and 7 (receptive and expressive communication) consistently group together regardless of setting and statistical method used. Furthermore, items 14 (Domestic activities) and 15 (Activities of daily living in the community), routinely cluster together and also form part of this stable subset of items in half of the proposed solutions. Less consistent but nonetheless noteworthy pairings are items 10 (sleep) with 11 (appetite) and items 1 (Behavioural concerns directed at others) and 3 (Other psychological and behavioural concerns).

**Table 3.** Comparison of published HoNOS-LD structures.

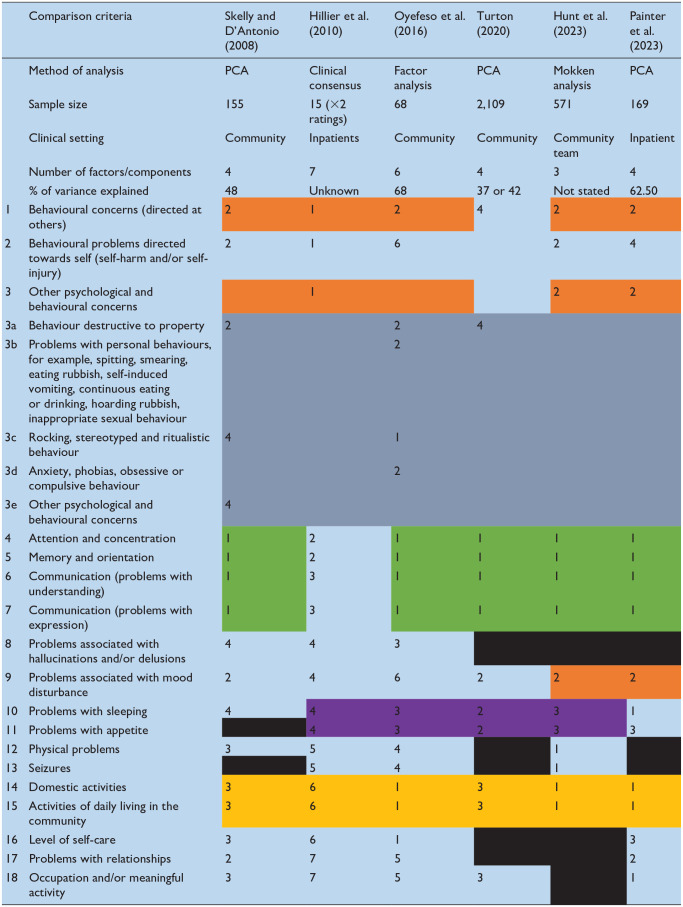							

Given the lack of a definitive solution, it also seems helpful to ascertain the internal consistency of each model. Having tested each published model on our dataset, Table 4 shows how the Cronbach’s alphas for each component compare. Whilst there is no absolute threshold for internal consistency, Taber (2018) provides a helpful summary of the levels that have been deemed acceptable in other studies. From that analysis, it seems that ±0.45 could be considered the boundary for an alpha, therefore results have been shaded accordingly.

**Table 4.** Comparison of each solution’s internal consistency (Cronbach’s alphas) when applied to our inpatient data set.

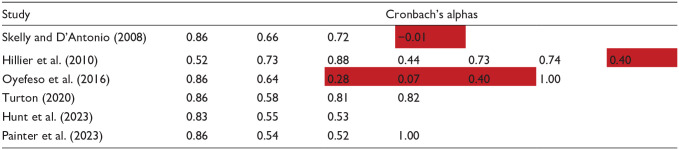							

Overall therefore, whilst each model has merit, three solutions have low alphas (Hillier et al., 2010; Oyefeso et al., 2016; Skelly & D’Antonio, 2008). Three would be problematic to apply at a national level due to the way they breach the tool’s rating guidance for item 3 (Oyefeso et al., 2016; Skelly & D’Antonio, 2008; Turton, 2020). Comparing the remaining solution (Hunt et al., 2023) with ours, there are differences in the setting (community vs. inpatient); the statistical methods (Mokken analysis vs. PCA); the items excluded (8, 16, 17 and 18 vs. 8, 12 and 13); the subsequent number of items included (14 vs. 15); and the number of groupings deemed optimal (3 vs. 4). Conversely, the two models have very similar levels of internal consistency. Equally there is a high degree of consistency in the items that have been grouped together, particularly given our fourth component is comprised of a single item.

### Limitations

As with any analysis of this type, our study has a number of limitations which must be borne in mind when considering the findings. The omission of three HoNOS-LD items from our model means some clinical improvements in some individuals (e.g. those with psychosis) may not be fully captured. However, PCA provides a more parsimonious method than tracking individual items and a more sensitive model than using total HoNOS-LD total scores alone. Our sample size was relatively modest, it was though the third largest of the published studies. Although ratings were all conducted by trained staff, they were not collected in highly controlled research settings, instead being drawn from routine clinical practice. However, this could be argued to have improved ecological validity. Finally, this was an exploratory analysis that still requires a confirmatory step, ideally with a different set of ratings (Orçan, 2018).

Despite these limitations, as well as the obvious confirmatory analysis mentioned above, based on our findings, a number of other recommendations can be made. All future studies should seek to gather and analyse a larger set of HoNOS-LD data that include both inpatient and community ratings. Following identification of the optimal statistical solution, a wider clinical consensus should be sought regarding any legitimate adjustments to the item groupings, as well as to their titles. Once the optimal balance of statistical and clinical properties has been attained, the groupings should be used in a range of settings to measure change in presentations over time. Finally, it should be noted that the HoNOS-LD has recently been updated and that consideration should be given to gathering data with the new HoNOS-ID (Painter et al., 2023).

## Conclusion

Our item reduction analysis of the HoNOS-LD has identified a four-component solution that offers an acceptable balance of statistical robustness and clinical face validity. It provides advantages over most other models in terms of internal consistency and/or viability for use at a national level in the UK. It has sufficient similarity with the only other viable model to make future (confirmatory) studies into these proposed solutions worthwhile. A confirmatory analysis is now required with larger datasets. The views of key stakeholders should be sought, and the model’s ability to measure change over time should be explored before the model is incorporated into routine practice.
